# *Helicobacter pylori* and Early Vascular Aging: Endothelial Dysfunction, Arterial Stiffness, and Conditional Cardiovascular Vulnerability

**DOI:** 10.3390/cimb48070740

**Published:** 2026-07-21

**Authors:** Federica Fogacci, Giulia Fiorini, Cristina Scollo, Claudio Borghi, Dino Vaira, Arrigo Francesco Giuseppe Cicero

**Affiliations:** 1 Department of Medical Pharmacology, Medical Faculty, Ataturk University, Erzurum 25240, Turkey; 2 Hypertension and Cardiovascular Risk Research Center, Medical and Surgical Sciences Department, Alma Mater Studiorum University of Bologna, 40130 Bologna, Italy; claudio.borghi@unibo.it (C.B.); berardino.vaira@unibo.it (D.V.); arrigo.cicero@unibo.it (A.F.G.C.); 3 Cardiovascular Medicine Unit, Heart, Chest and Vascular Department, IRCCS Azienda Ospedaliero-Universitaria di Bologna, 40138 Bologna, Italy; cristina.scollo@aosp.bo.it

**Keywords:** *Helicobacter pylori*, endothelial dysfunction, arterial stiffness, vascular aging, flow-mediated dilation, pulse-wave velocity, cytotoxin-associated gene A (CagA), extracellular vesicles, oxidative stress

## Abstract

*Helicobacter pylori* (*H. pylori*) infection has been investigated as a potential contributor to extra-gastric vascular injury, although its cardiovascular relevance remains uncertain and context-dependent. This state-of-the-art narrative review synthesizes clinical, translational, and experimental evidence linking *H. pylori* infection to endothelial dysfunction and arterial stiffness, two complementary phenotypes of early vascular aging. Evidence is strongest for endothelial dysfunction, particularly in the presence of active or cytotoxin-associated gene A (CagA)-positive infection, extracellular-vesicle-mediated signaling, oxidative stress, impaired endothelial repair, selective attenuation of endothelium-dependent vasodilation, and short-term improvement after eradication. Associations with arterial stiffness are less consistent and appear more evident in selected settings characterized by younger age, inflammatory or metabolic vulnerability, and severe gastric injury. Conversely, serology-based studies and studies using late structural vascular endpoints frequently report null or discordant findings. Overall, *H. pylori* should not be considered a universal cardiovascular risk factor, but it may amplify early vascular injury in susceptible subgroups. Prospective studies using active-infection testing, virulence profiling, gastric histology, and prespecified vascular endpoints are needed to determine whether eradication produces sustained vascular benefit.

## 1. Introduction

*Helicobacter pylori* (*H. pylori*) has long been regarded primarily as a gastroduodenal pathogen, but its effects may extend beyond the gastric mucosa in selected clinical contexts [1,2]. Its cardiovascular relevance, however, remains controversial. Gastric colonization by itself rarely leads to increased vascular risk. Systemic effects may arise when factors such as bacterial virulence, host susceptibility, metabolic stress, inflammation, and gastric injury interact to disrupt vascular homeostasis [3,4,5,6]. This interpretation is consistent with the contemporary view of vascular disease as a progressive process rather than the consequence of a single isolated exposure [7,8]. Atherosclerosis and vascular aging develop through prolonged preclinical phases in which endothelial dysfunction, oxidative stress, immune activation, arterial stiffening, and microvascular injury precede overt cardiovascular events [7,8,9,10]. Vascular aging refers to the progressive loss of endothelial homeostasis and arterial elasticity, accompanied by increased arterial stiffness, oxidative stress, inflammation, and structural remodeling [7,8,9,10]. When these alterations occur earlier or progress more rapidly than expected for chronological age, they are commonly described as early vascular aging [7,8,9,10]. In this framework, the relevant question is not whether *H. pylori* directly causes cardiovascular disease, but whether it can amplify early vascular injury in susceptible individuals [2,11,12].

Endothelial dysfunction represents the most plausible initial interface of *H. pylori*-related injury [5,13,14]. As an early, largely nitric oxide-dependent, and potentially reversible disturbance of vascular homeostasis, it is particularly suited to capture infection-related functional damage [7,8,9]. Accordingly, reduced flow-mediated dilation (FMD), impaired acetylcholine-mediated relaxation, preserved nitroglycerin responsiveness, and improvement after eradication define a pattern more consistent with selective endothelial injury than with nonspecific vascular impairment [5,13,14].

Arterial stiffness may represent a later stage of the same continuum. Unlike endothelial dysfunction, which captures a dynamic and potentially reversible abnormality, arterial stiffness reflects structural and functional arterial-wall remodeling, including extracellular-matrix reorganization, elastin fragmentation, collagen deposition, medial calcification, vascular-smooth-muscle-cell dysfunction, oxidative stress, and chronic inflammation [10,15,16]. For this reason, its association with *H. pylori* is expected to be less uniform and more dependent on age, blood pressure load, metabolic disease, inflammatory tone, and gastric-disease burden [11,17,18,19].

A useful conceptual framework is therefore one of selective susceptibility rather than universal cardiovascular causality [2,11,12]. *H. pylori* may become clinically relevant when infection is active, strain virulence is high, or the patient profile is already shaped by inflammatory, metabolic, or gastric pathology [12,19,20,21,22,23,24,25]. In this view, the stomach is not merely the anatomical site of infection, but a potential source of immune, metabolic, and extracellular-vesicle-mediated signals capable of influencing systemic vascular biology [5,6,10].

This review critically examines the evidence linking *H. pylori* infection with two complementary phenotypes: endothelial dysfunction and arterial stiffness. Rather than treating positive and negative studies as mutually incompatible, we interpret the literature according to the sensitivity of the vascular endpoint, and the clinical context in which the association was observed.

## 2. Methods

### 2.1. Review Design and Scope

This article was designed as a state-of-the-art narrative review aimed at critically synthesizing the clinical, translational, and experimental evidence linking *H. pylori* infection to early vascular injury, with a specific focus on endothelial dysfunction and arterial stiffness. The review was not conceived as a systematic review or meta-analysis; therefore, no formal protocol was registered, and no quantitative evidence synthesis or formal risk-of-bias assessment was performed. Nevertheless, a structured literature-search and study-selection approach was adopted to improve methodological transparency and reproducibility.

### 2.2. Literature Search Strategy

A targeted literature search was conducted in PubMed/MEDLINE, Scopus, Web of Science, and Google Scholar from database inception through May 2026. The search strategy combined terms related to *H. pylori* infection with terms related to endothelial function, arterial stiffness, vascular aging, bacterial virulence, and eradication therapy. Search terms included combinations of “*Helicobacter pylori*” or “*H. pylori*” with “endothelial dysfunction”, “flow-mediated dilation”, “FMD”, “arterial stiffness”, “pulse-wave velocity”, “PWV”, “brachial–ankle pulse-wave velocity”, “baPWV”, “cardio–ankle vascular index”, “CAVI”, “vascular aging”, “CagA”, “cytotoxin-associated gene A”, and “eradication therapy”. Reference lists of relevant original studies and reviews were also manually screened to identify additional eligible publications.

### 2.3. Eligibility Criteria

Eligible publications comprised original clinical studies, including cohort, case-control, interventional, and pre- and post-eradication studies, as well as experimental and translational investigations, pertinent reviews, and commentaries addressing *H. pylori* infection in relation to endothelial function, arterial stiffness, vascular aging, immune activation, oxidative stress, or infection-related vascular injury.

Priority was given to studies that directly evaluated *H. pylori* infection or gastric injury together with vascular-function endpoints, including FMD, pulse-wave velocity (PWV), brachial–ankle PWV (baPWV), estimated PWV, cardio–ankle vascular index (CAVI), vascular biomarkers, or vascular changes following eradication therapy. Studies were considered particularly informative when the infectious exposure was characterized using active-infection testing, bacterial virulence profiling, gastric histology, infection severity, or documented eradication response. Both studies reporting positive associations and studies reporting null or inconsistent findings were considered relevant to the review.

Publications were excluded when they did not address *H. pylori* infection or vascular outcomes relevant to the scope of the review, focused exclusively on gastrointestinal disease without systemic vascular or cardiometabolic endpoints, provided insufficient methodological or outcome information, were available only as conference abstracts without full-text data, or represented duplicate publications. When multiple reports appeared to include overlapping study populations, the most complete or methodologically informative publication was prioritized.

### 2.4. Study Selection and Data Extraction

Titles and abstracts identified through the literature search were screened for relevance to the review question. Potentially eligible articles were subsequently assessed in full text against the eligibility criteria described above. Studies were considered relevant when they evaluated *H. pylori* infection or gastric injury in relation to endothelial dysfunction, arterial stiffness, vascular aging, cardiometabolic vulnerability, or related mechanistic pathways, irrespective of whether the reported association was positive, negative, or inconclusive.

For each eligible study, relevant information was extracted regarding study design, population characteristics, method of *H. pylori* or gastric-injury assessment, vascular endpoint, main findings, adjustment for potential confounders, and interpretive relevance. Particular attention was paid to whether infection was defined by serology alone or by biologically more informative measures, including urea breath testing, biopsy, histology, cytotoxin-associated gene A (CagA) status, gastric inflammatory severity, atrophic gastritis, intestinal metaplasia, or documented vascular response following eradication.

### 2.5. Evidence Synthesis

Because of heterogeneity in study design, infection assessment, vascular endpoints, populations, follow-up duration, and adjustment strategies, findings were synthesized narratively rather than quantitatively. The evidence was organized according to vascular phenotype, distinguishing endothelial dysfunction from arterial stiffness, and according to the biological informativeness of the methods used to assess infection or gastric injury. Experimental and translational studies were considered separately from clinical studies and were used to evaluate mechanistic plausibility, including CagA-dependent signaling, extracellular-vesicle transfer, oxidative stress, endothelial-repair impairment, inflammatory endothelial programming, and sex-specific vascular susceptibility.

The synthesis considered the consistency of findings across study designs, the specificity of vascular endpoints, the definition of infectious exposure, reversibility following eradication, and the clinical contexts in which positive, negative, or inconsistent associations were observed. Given the narrative design and the predominantly observational nature of the clinical evidence, the conclusions were framed cautiously and should be regarded as hypothesis-generating rather than as definitive evidence of causality.

## 3. The Endothelium as the First Vascular Interface of *Helicobacter Pylori*

The endothelium provides the most biologically plausible vascular interface through which *H. pylori* may exert systemic effects, because endothelial homeostasis is highly responsive to inflammatory, oxidative, metabolic, and extracellular-vesicle-mediated signals [7,8,10]. In contrast to carotid intima-media thickness or coronary calcification, which reflect relatively late and cumulative structural remodeling, endothelial function captures an earlier, dynamic, and potentially reversible stage of vascular injury [5,9]. The most informative *H. pylori* studies go beyond measuring seropositivity or structural atherosclerotic changes. They focus on demonstrating selective impairment of endothelium-dependent vasodilation. This is especially meaningful when endothelium-independent smooth-muscle responsiveness is preserved, or when vascular function improves after eradication of the infection [5,13,14].

### 3.1. Functional Evidence: Selectivity, Reversibility, and Early Vascular Injury

The clinical evidence is most persuasive when two conditions are met: the vascular phenotype is functional rather than structural, and the infectious exposure reflects biological activity rather than remote serological contact [3,5]. This principle is illustrated by coronary physiology studies in which cumulative pathogen burden associates with coronary artery disease, with an odds ratio (OR) of approximately 1.3 per pathogen, but its more important implication is mechanistic rather than epidemiologic [13]. The vascular abnormality localizes predominantly to the endothelium, because pathogen burden tracks with acetylcholine-mediated endothelial dysfunction while responses to sodium nitroprusside and adenosine remain preserved [13]. Thus, the central signal is not simply that infection burden accompanies coronary disease, but that chronic infectious exposure identifies a selective disturbance of endothelial vasomotor regulation [13].

Active *H. pylori* infection provides a more direct test of this concept [5]. In young adults, infection is associated with reduced FMD, while experimental models reproduce the same biological pattern through impaired acetylcholine-mediated relaxation and preserved nitroglycerin-mediated responsiveness [5]. The improvement in endothelial function after eradication is pivotal, because it moves the observation beyond cross-sectional association and supports a potentially reversible infection-related vascular phenotype [5]. Pediatric data extend this sequence to an earlier stage of vascular vulnerability, showing substantially lower FMD in infected children, approximately 6% compared with 9.5–10% in comparator groups, together with improvement after eradication and parallel changes in inflammatory and lipid indices [14]. The correlation between FMD and gastric inflammatory severity further suggests that endothelial impairment may reflect biological burden rather than infection status alone [14]. However, the clinical evidence regarding vascular improvement after eradication remains preliminary and is based on two small, non-randomized pre/post studies. In one, baseline FMD was assessed in 18 infected participants and 13 controls, while only 10 treated patients underwent post-eradication reassessment [5]. In the other, 30 infected children were compared with 30 *H. pylori*-negative symptomatic children and 30 healthy controls, with vascular reassessment performed 3 months after a 2-week eradication regimen [14]. Although both studies reported improvement in endothelial function after successful eradication, the limited post-eradication sample sizes and short follow-up preclude conclusions regarding the durability of the vascular benefit. Moreover, the available data do not establish whether the observed improvement was independent of concurrent changes in systemic inflammation or lipid profile, and neither study assessed long-term arterial-stiffness progression or cardiovascular outcomes.

Negative studies materially limit the strength and generalizability of the endothelial hypothesis [3,4]. In healthy young men without cardiovascular risk factors, *H. pylori* seropositivity and cumulative infectious burden were not associated with brachial FMD, despite the assessment of both endothelium-dependent and endothelium-independent vascular responses [3]. This finding indicates that serological exposure alone does not identify a reproducible endothelial phenotype in low-risk populations. Differences in infection ascertainment, strain virulence, inflammatory activity, or host susceptibility may partly explain this null result; however, the possibility that *H. pylori* has no clinically meaningful vascular effect in such populations must also be acknowledged [3,5,26].

Microvascular observations add a plausible but less definitive metabolic extension of the endothelial hypothesis [27,28]. In slow coronary flow, *H. pylori* infection coexists with higher homocysteine, lower folate, and a higher TIMI frame count, suggesting a possible link among gastric infection, one-carbon metabolism, and coronary microvascular dysfunction [27]. This pathway should not be generalized, however, because cardiac syndrome X studies do not show a significant difference in homocysteine according to *H. pylori* status within the syndrome [28]. Homocysteine-related mechanisms may therefore represent a context-specific modifier rather than a universal mediator of *H. pylori*-associated endothelial dysfunction [27,28].

Overall, the functional evidence suggests a potentially relevant but conditional endothelial phenotype. Positive associations are more frequently observed when vascular assessment captures early and potentially reversible dysfunction and when infection is characterized by measures of biological activity rather than serology alone [3,5,13,14]. However, the limited number of studies, the heterogeneity of infection assessment, and the presence of null findings prevent firm conclusions regarding the consistency or generalizability of this association.

### 3.2. Mechanistic Convergence: CagA, Exosomes, Oxidative Stress, and Endothelial Repair

The evidence discussed in this section derives predominantly from experimental and translational models and should be interpreted as supporting biological plausibility rather than as establishing a causal vascular effect in humans. A central mechanistic issue is how a pathogen largely confined to the gastric mucosa can influence distant vascular beds. The most persuasive explanation is that *H. pylori* does not require direct vascular invasion to alter endothelial biology but may instead act through gastric-derived extracellular vesicles carrying bacterial virulence signals [5,6]. In this model, CagA-positive *H. pylori* induces gastric epithelial cells to release CagA-containing exosomes that are internalized by endothelial cells and impair migration, proliferation, and tube formation, thereby linking a mucosal infection to defective endothelial repair [5]. Serum exosomes from infected subjects and experimental animals reproduce endothelial dysfunction in vitro, whereas pharmacological inhibition of exosome release preserves endothelial function in infected mice, supporting extracellular-vesicle transfer as a biologically active mediator rather than an epiphenomenon [5].

Redox biology provides the second point of mechanistic convergence. CagA-positive, but not CagA-negative, infection increases aortic reactive oxygen species, impairs acetylcholine-mediated relaxation, and promotes early atherosclerotic changes in experimental models [6]. The prevention of these abnormalities by antioxidant treatment and by inhibition of exosome release places CagA-containing exosomes and oxidative stress upstream of endothelial dysfunction, and integrates microbial virulence, vesicular communication, redox imbalance, and vascular phenotype specificity into a single causal sequence [5,6].

This exosomal-redox pathway is complemented by inflammatory endothelial programming. In endothelial-cell systems and infected animals, *H. pylori* activates a GATA3–CHI3L1–p38 MAPK axis associated with impaired proliferation, migration, and tube formation [29]. The attenuation of this phenotype after *GATA3* knockdown indicates that endothelial injury is not merely the passive consequence of oxidative stress, but also reflects regulated transcriptional and kinase-dependent inflammatory signaling [29].

Sex-specific experimental data further refine this model. In CagA-positive infection, male mice develop endothelial dysfunction and increased aortic reactive oxygen species, whereas female mice appear relatively protected [24]. Exosomes from infected male animals impair endothelial repair phenotypes in vitro, and antioxidant treatment prevents these effects, suggesting that sex may modify the vascular consequences of CagA-driven oxidative signaling [24]. This observation is clinically relevant because human stiffness studies also indicate stronger vascular associations in men, particularly when inflammatory activation is present [19,24].

**Figure 1:**
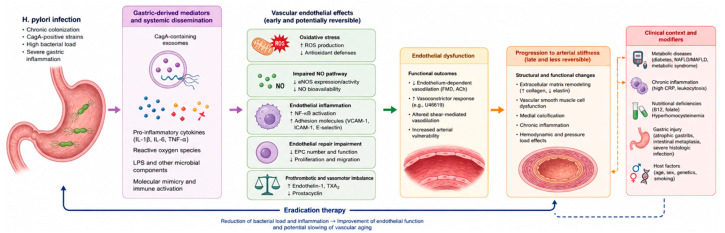
Pathophysiological mechanisms linking *Helicobacter pylori* infection to vascular dysfunction, including extracellular-vesicle signaling, oxidative stress, inflammatory endothelial programming, impaired endothelial repair, and arterial remodeling. Abbreviations: ACh, acetylcholine; CagA, cytotoxin-associated gene A; CRP, C-reactive protein; eNOS, endothelial nitric oxide synthase; EPC, endothelial progenitor cells; FMD, flow-mediated dilation; *H. pylori*, *Helicobacter pylori*; ICAM-1, intercellular adhesion molecule 1; IL-1β, interleukin 1 beta; IL-6, interleukin 6; LPS, lipopolysaccharide; MASLD, metabolic dysfunction-associated steatotic liver disease; NAFLD, nonalcoholic fatty liver disease; NF-κB, nuclear factor kappa B; NO, nitric oxide; ROS, reactive oxygen species; TNF-α, tumor necrosis factor alpha; TXA_2_, thromboxane A_2_; U46619, thromboxane A_2_ analog; VCAM-1, vascular cell adhesion molecule 1; ↑, increased; ↓, decreased.

The mechanistic literature therefore supports a biologically integrated model in which virulent *H. pylori* strains generate gastric-derived vesicular signals that reach the endothelium, increase oxidative stress, impair endothelial repair, activate inflammatory pathways, and selectively compromise endothelium-dependent vasodilation [5,6,24,29]. This mechanistic convergence provides the strongest biological rationale for considering endothelial dysfunction as the primary vascular interface of *H. pylori* infection [5,13,14] (Figure 1).

### 3.3. Why the Endothelial Signal Is Stronger than the Structural Signal

Studies using structural vascular endpoints have not demonstrated a consistent association between *H. pylori* seropositivity and established atherosclerosis [4,17,30]. In the Multi-Ethnic Study of Atherosclerosis (MESA), neither *H. pylori* seropositivity nor cumulative pathogen burden was associated with carotid intima-media thickness or coronary artery calcification [4]. Other studies conducted in patients at increased cardiovascular risk have similarly shown limited or inconsistent relationships between *H. pylori* antibodies and structural vascular measures [17,30]. Taken together, these findings argue against *H. pylori* as a universal or major independent determinant of atherosclerotic burden.

Functional and structural vascular endpoints may nevertheless capture different stages of vascular injury. FMD and acetylcholine-mediated vasorelaxation assess dynamic and potentially reversible abnormalities, whereas carotid intima-media thickness and coronary artery calcification reflect cumulative arterial remodeling influenced by age, blood pressure, lipid and glycemic burden, renal function, smoking, diet, and socioeconomic factors [4,5,9,30]. This difference offers one possible explanation for the heterogeneous findings, but it does not establish that negative structural studies represent false-negative results or that endothelial dysfunction necessarily precedes structural vascular disease in individuals with *H. pylori* infection. Such a temporal model remains biologically plausible but requires prospective validation.

**Figure 2:**
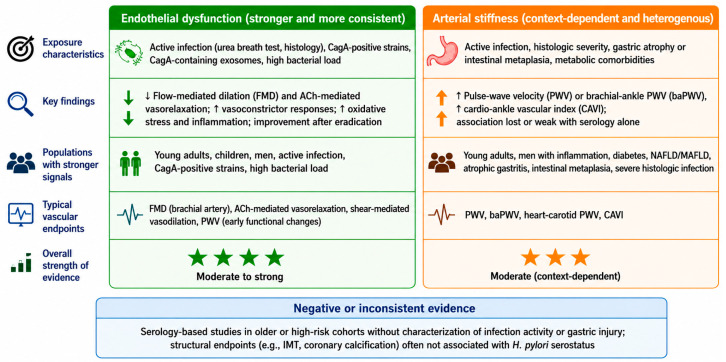
Evidence landscape linking *Helicobacter pylori* infection to endothelial dysfunction and arterial stiffness. Abbreviations: ACh, acetylcholine; baPWV, brachial–ankle pulse-wave velocity; CagA, cytotoxin-associated gene A; CAVI, cardio-ankle vascular index; FMD, flow-mediated dilation; *H. pylori*, *Helicobacter pylori*; MAFLD, metabolic dysfunction-associated fatty liver disease; NAFLD, non-alcoholic fatty liver disease; PWV, pulse-wave velocity.

The positive evidence appears more coherent when active infection, gastric inflammatory severity, virulence characteristics, eradication response, and functional vascular endpoints are considered [3,4,5,6,14]. However, these observations remain context-dependent and should be interpreted as hypothesis-supporting rather than as definitive evidence of a causal vascular effect. The absence of consistent associations in serology-based and structural-imaging studies represents an important limitation of the proposed relationship rather than merely a consequence of less informative exposure or outcome assessment (Figure 2).

A rigorous interpretation of the endothelial literature therefore requires attention to both sides of the exposure–endpoint relationship. On the exposure side, active infection, virulence profiling, eradication response, and gastric inflammatory severity are more informative than antibody status alone [5,14,26]. On the endpoint side, FMD and acetylcholine-mediated vasorelaxation are better suited to detecting early endothelial injury than carotid intima-media thickness or coronary calcium [4,5,9]. The endothelial signal becomes most persuasive when these biologically meaningful exposure definitions are paired with sensitive functional vascular readouts (Table 1). The principal experimental and translational evidence supporting these mechanisms is summarized in Table 2.

## 4. Arterial Stiffness as the Vascular-Aging Readout of Chronic Infection

Arterial stiffness represents a more integrated and temporally accumulated vascular phenotype than endothelial dysfunction. Whereas endothelial dysfunction reflects an early and potentially reversible disturbance of vascular homeostasis, arterial stiffness embodies progressive remodeling of the arterial wall through extracellular-matrix reorganization, vascular-smooth-muscle-cell dysfunction, elastin fragmentation, collagen accumulation, medial calcification, inflammation, and altered central hemodynamics [10,15,16]. For this reason, *H. pylori*-related stiffness is expected to be harder to detect than endothelial impairment and more dependent on the duration of exposure, vascular aging, blood pressure load, metabolic disease, and gastric inflammatory burden [11,18,19,21].

### 4.1. Serology as an Imperfect Vascular Exposure

The arterial-stiffness literature does not support a simple or consistent relationship between *H. pylori* seropositivity and arterial remodeling [11,17]. In populations at increased cardiovascular risk, antibodies against *H. pylori* were not consistently associated with carotid intima-media thickness, carotid stenosis, or carotid elastic pressure modulus [17]. These null findings may partly reflect the inability of serology to distinguish remote exposure from active infection, but they also indicate that a broadly generalizable and independent vascular association has not been established [3,4,17].

Similarly, cumulative pathogen burden and individual pathogen serostatus have not shown consistent associations with carotid intima-media thickness or coronary artery calcification [4,30]. More informative associations have been reported in selected studies using active-infection testing, gastric histology, or measures of infection severity [21,22,25]. However, these findings derive from specific populations and require independent replication; they should not be interpreted as resolving or invalidating the negative evidence obtained in serology-based cohorts.

### 4.2. Age, Sex, and Inflammation as Biological Filters

Age, sex, and inflammatory tone appear to function as biological filters that determine whether an *H. pylori*-associated stiffness signal becomes detectable. In a Japanese screening cohort, seropositivity is accompanied by a modest inflammatory–metabolic profile, including lower high-density lipoprotein cholesterol [55.0 vs. 58.0 mg/dL; *p* < 0.0001] and higher leukocyte count [5907 vs. 5504 × 10^9^/L; *p* < 0.0001] [18]. Although overall PWV does not differ by serostatus, heart–carotid PWV is higher in seropositive individuals younger than 39 years [632.2 ± 15.7 vs. 589.7 ± 10.6 cm/s; *p* = 0.027], whereas this difference is no longer evident with advancing age [18]. This age-dependent pattern suggests that infection-related arterial changes may be more discernible before chronological aging and pressure load become the dominant determinants of arterial stiffness [11,18].

Sex and inflammation further sharpen this phenotype. In men, *H. pylori* seropositivity is independently associated with high baPWV [OR, 1.27; 95% confidence interval (CI), 1.05–1.52], and the association becomes stronger when seropositivity coexists with C-reactive protein >0.045 mg/dL [OR, 1.50; 95% CI, 1.14–1.98] [19]. The signal is most pronounced in men aged ≤49 years, in whom combined seropositivity and elevated C-reactive protein confers a higher likelihood of increased stiffness [OR, 1.81; 95% CI, 1.16–2.80], while no independent association is evident in older men or in women [19]. This clinical pattern is consistent with experimental evidence showing male-selective endothelial oxidative injury after CagA-positive infection, suggesting that sex-related vascular susceptibility may modulate the arterial consequences of chronic gastric infection [24].

### 4.3. Biological Burden Beyond Antibody Status

The vascular signal becomes more informative when the exposure is defined by biological burden rather than by antibody status alone. In asymptomatic adults, *H. pylori* seropositivity is independently associated with high CAVI [adjusted OR, 1.36; 95% CI, 1.10–1.68; *p* = 0.005], suggesting that stiffness-related associations may be detectable when the vascular phenotype is assessed with an index less dependent on instantaneous blood pressure than conventional PWV [31]. Nevertheless, the most conceptually compelling evidence comes from histology-based assessment, where the severity of gastric infection, rather than seropositivity alone, defines the biological exposure [25].

In young adults with histologically confirmed infection, estimated PWV (ePWV) is higher in infected than uninfected participants [6.66 ± 0.60 vs. 6.33 ± 0.58 m/s; *p* < 0.001] [25]. More importantly, ePWV increases across Updated Sydney System severity categories, and severe infection is associated with a markedly higher likelihood of elevated ePWV [OR, 3.87; 95% CI, 3.25–4.60; *p* < 0.001] [25]. This graded relationship suggests that arterial stiffening may reflect the biological intensity of gastric infection more closely than the mere presence of circulating antibodies [25].

### 4.4. Metabolic Vulnerability as a Vascular Amplifier

Metabolic disease appears to make the vascular consequences of *H. pylori* more visible, not because infection becomes an isolated cause of arterial stiffness, but because it acts on an arterial wall already primed by insulin resistance, glycation, oxidative stress, endothelial dysfunction, inflammation, and pressure-related remodeling [15,16,20,23]. In type 2 diabetes, infected patients have higher PWV than uninfected patients [1877 ± 550 vs. 1585 ± 331 cm/s; *p* = 0.0005], and infection remains an independent determinant of PWV after adjustment for major hemodynamic covariates [β = 0.169; *p* = 0.0220] [20]. This association becomes more pronounced in a larger diabetic cohort using active-infection testing, in which infected subjects show substantially higher baPWV [2031.61 ± 525.48 vs. 1556.68 ± 227.54 cm/s; *p* < 0.01] and a higher prevalence of severe peripheral arterial stiffness [62.7% vs. 21.9%; *p* < 0.01] [23]. These findings position diabetes as a vascular amplification state in which chronic gastric infection may intensify, rather than independently initiate, arterial aging [20,23].

A similar interaction emerges in metabolic liver disease [12]. In asymptomatic individuals, nonalcoholic fatty liver disease combined with *H. pylori* infection confers higher odds of increased arterial stiffness [OR, 2.23; 95% CI, 1.63–3.06] than nonalcoholic fatty liver disease without infection [OR, 1.61; 95% CI, 1.15–2.26] [12]. Among individuals with metabolic dysfunction-associated fatty liver disease, *H. pylori* infection additively increases stiffness risk [OR, 2.13; 95% CI, 1.64–2.78], supporting convergence among gastric infection, hepatic–metabolic dysfunction, insulin resistance, systemic inflammation, and arterial-wall remodeling [12]. This interpretation is consistent with commentary proposing active *H. pylori* infection as a potential modifier of the relationship among metabolic syndrome, fatty liver disease, arterial stiffness, and cardio–cerebrovascular risk [32].

### 4.5. Gastric Injury as a Marker of Cumulative Vascular Exposure

Cumulative gastric injury may provide a more informative vascular exposure than infection status alone. In older adults, pepsinogen-defined atrophic gastritis is associated with higher adjusted baPWV [16.63 ± 3.50 vs. 15.59 ± 3.47 m/s; *p* = 0.010] and higher CAVI [8.59 ± 1.20 vs. 8.27 ± 1.19; *p* = 0.022], whereas *H. pylori* immunoglobulin G status alone shows only nonsignificant trends [21]. This distinction is biologically important because atrophic gastritis may mark prolonged gastric inflammation, cumulative microbial exposure, altered acid secretion, and nutritional disturbance even when serology becomes less informative [21].

Histologic gastric lesions reinforce the same concept. Patients with gastric intestinal metaplasia or atrophic gastritis have higher PWV than controls [10.07 ± 1.79 and 9.85 ± 1.84 vs. 8.99 ± 1.95 m/s], and PWV >10 m/s is substantially more frequent in both gastric-injury groups than in controls [49.5%, 47.8%, and 18.8%; *p* < 0.001] [22]. Low vitamin B12, gastric intestinal metaplasia, and atrophic gastritis independently predict elevated PWV, suggesting that malabsorption, micronutrient deficiency, homocysteine-related vascular stress, and chronic gastric inflammation may contribute to arterial stiffening [22].

This evidence reframes gastric pathology as a cumulative biological record rather than a purely local consequence of infection. In vascular terms, atrophic gastritis, intestinal metaplasia, vitamin B12 disturbance, and histologic infection severity may capture the duration and systemic consequences of the host–pathogen interaction more effectively than antibody status alone [21,22,25].

The principal clinical and contextual evidence linking *H. pylori* infection or gastric injury to arterial stiffness is summarized in Table 3.

### 4.6. Experimental Bridge and Interpretive Boundaries

Experimental evidence helps connect endothelial injury with later arterial remodeling, particularly when infection is studied in the presence of metabolic stress. In a guinea-pig model, persistent *H. pylori* infection combined with a high-fat diet increases systemic inflammation and reduces pulse-wave amplitude, a pattern consistent with impaired arterial elasticity [34]. The relevance of this model lies less in direct clinical extrapolation than in its biological convergence with human data, in which the vascular signal of infection becomes most apparent when metabolic vulnerability is present [12,34].

The boundaries of this model are equally important. Not all systemic consequences of *H. pylori* appear to be mediated by arterial stiffness, and infection-related risk may involve pathways that are not captured by PWV [33]. In a nested case–control study, *H. pylori* seropositivity was associated with cognitive decline in the final stepwise multivariable model [OR, 4.468; 95% CI, 1.535–13.00], whereas PWV was not retained as an independent predictor, suggesting that systemic immune or metabolic disruption may extend beyond measurable arterial stiffening [33]. More broadly, the clinical literature supports biological plausibility but does not establish the direction or causality of the reported associations. Most available studies are cross-sectional, case–control, or observational, and therefore cannot determine whether *H. pylori* infection preceded the development of endothelial dysfunction or arterial stiffness. Reverse causation and bidirectional relationships cannot be excluded, because individuals with cardiometabolic disease or established vascular impairment may differ in healthcare utilization, likelihood of undergoing gastrointestinal testing, dietary patterns, nutritional status, gastric pathology, and medication exposure, all of which may influence both infection ascertainment and vascular measurements [2,11,35].

Residual confounding also remains an important concern. Age, smoking, adiposity or body mass index, blood pressure, diabetes, renal function, lipid profile, diet, physical activity, socioeconomic conditions, and concomitant medications are associated with vascular function and may also correlate with the acquisition, persistence, diagnosis, or treatment of *H. pylori* infection. Although several studies adjusted for selected cardiovascular risk factors, the covariates considered and the methods used to measure them varied substantially, and some relevant factors were unavailable or incompletely captured. Reported adjusted associations should therefore not be interpreted as evidence that *H. pylori* independently causes vascular dysfunction.

A systematic review and meta-analysis of 24 observational studies reported higher CIMT and PWV and lower FMD in *H. pylori*-positive individuals; however, the FMD analysis included only two studies and between-study heterogeneity was very high, limiting the certainty and generalizability of the pooled estimates [36].

## 5. Integrated Pathophysiological Model

The available evidence supports a model in which *H. pylori* contributes to vascular dysfunction only when microbial, host, metabolic, and gastric-disease determinants converge [2,3,4,11]. Within this framework, the endothelium represents the earliest and most coherent vascular interface of infection, because active CagA-positive strains can generate gastric-derived exosomal signals, increase reactive oxygen species, impair nitric oxide-dependent vasodilation, disrupt endothelial repair, and activate inflammatory signaling pathways [5,6,29]. These mechanisms closely overlap with established pathways of endothelial dysfunction, including oxidative stress, impaired nitric oxide signaling, inflammatory activation, altered mechanotransduction, thrombogenicity, senescence, and defective vascular repair [7,8,9].

Arterial stiffness occupies a later position in this biological sequence. It may represent the stage at which repeated endothelial injury is incorporated into extracellular-matrix remodeling, vascular-smooth-muscle-cell dysfunction, altered central hemodynamics, and progressive arterial-wall aging [10,15,16]. Conditions characterized by insulin resistance, chronic inflammation, micronutrient disturbance, or advanced gastric pathology may facilitate this transition, making the vascular consequences of *H. pylori* more detectable in metabolically or histologically enriched populations [12,20,21,22,23,25].

This framework also helps explain the heterogeneity of clinical literature. Serology-based studies may underestimate relevant vascular exposure, whereas older cohorts may be dominated by age- and pressure-related arterial remodeling that obscures smaller infection-related effects [3,4,11,17,18]. By contrast, vascular signals are more likely to emerge in younger individuals, in men with inflammatory activation, in patients with cardiometabolic disease, and in cohorts characterized by active infection, gastric histology, or infection severity [5,12,14,19,23,24,25].

**Figure 3:**
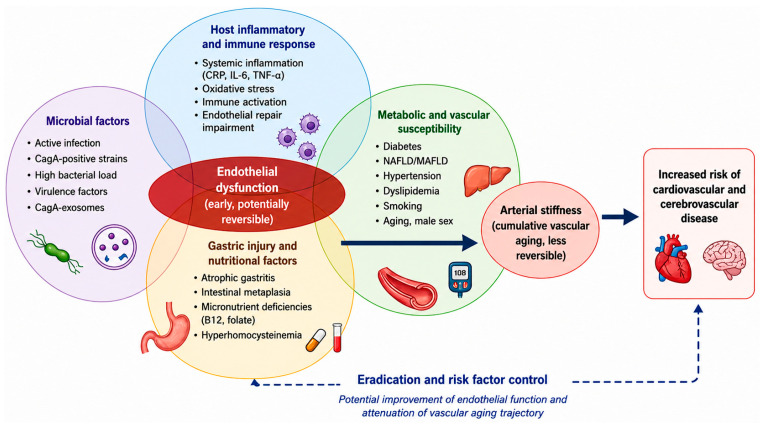
Integrated model: *Helicobacter pylori* as a conditional amplifier of early vascular aging. Abbreviations: B12, vitamin B12; CagA, cytotoxin-associated gene A; CRP, C-reactive protein; IL-6, interleukin 6; MAFLD, metabolic dysfunction-associated fatty liver disease; NAFLD, nonalcoholic fatty liver disease; TNF-α, tumor necrosis factor alpha.

More broadly, *H. pylori* fits within an emerging immunovascular paradigm in which chronic mucosal infection may influence systemic vascular aging through persistent antigenic stimulation, immune remodeling, extracellular-vesicle signaling, oxidative stress, and post-infectious vascular injury [10,37,38]. Its relevance is therefore not that of an isolated cardiovascular pathogen, but that of a chronic biological stressor whose vascular effects become manifest when microbial activity and host susceptibility intersect [5,6,10] (Figure 3).

## 6. Clinical and Research Implications

The available evidence does not support *H. pylori* testing as a cardiovascular screening or risk-stratification strategy in unselected populations [3,4,17]. It also does not justify eradication therapy solely for the prevention or treatment of endothelial dysfunction or arterial stiffness. Accordingly, the present evidence should not modify established clinical indications for *H. pylori* testing and eradication. The potential vascular relevance of the infection should therefore be investigated primarily in research settings and in carefully phenotyped high-risk cohorts, using endpoints such as FMD, acetylcholine-mediated vasorelaxation, PWV, and CAVI [5,9,25,31].

The next generation of studies should therefore move beyond binary serology as a screening test and adopt a multidimensional exposure framework in which the detection of an active infection is needed. Active-infection testing, CagA and virulence profiling, gastric histologic grading, sex-stratified analyses, metabolic phenotyping, inflammatory biomarkers, and prespecified vascular endpoints should be incorporated into prospective and interventional designs [5,9,11,26]. Randomized eradication trials with prespecified endothelial and arterial-stiffness outcomes will be essential to determine whether *H. pylori* is only a marker of vascular vulnerability or a modifiable contributor to early vascular aging, and will be required before testing or eradication can be recommended for cardiovascular prevention [5,14].

## 7. Limitations

Publication bias and selective outcome reporting cannot be excluded. Some of the positive findings derive from relatively small, single-center observational, pre/post-eradication, or mechanistic studies, whereas null or inconclusive results may be less likely to be published or emphasized. In addition, despite the adoption of a structured literature-search strategy, the narrative design of this review entails a potential risk of selective study inclusion or interpretive emphasis. Because of the substantial heterogeneity in infection assessment, vascular endpoints, study populations, follow-up duration, and analytical methods, a formal assessment of publication bias was not feasible. Consequently, the apparent coherence of the positive evidence may overestimate the magnitude, reproducibility, or clinical relevance of the association between *H. pylori* infection and vascular dysfunction.

Most of the available clinical evidence is observational, predominantly cross-sectional or case–control, and therefore cannot establish temporality or causality. Comparability across studies is further limited by substantial heterogeneity in study populations, methods used to assess *H. pylori* exposure, vascular endpoints, adjustment strategies, and follow-up duration. In particular, serology, active-infection testing, bacterial virulence profiling, and gastric histology do not represent equivalent exposure measures, while FMD, PWV, baPWV, CAVI, ePWV, and structural vascular markers capture different vascular phenotypes. The limited number and sample size of eradication studies, together with the absence of long-term vascular outcomes, further restrict causal interpretation. Accordingly, the conclusions of this review should be regarded as hypothesis-generating rather than definitive.

The predominantly observational nature of the clinical evidence limits causal interpretation. Cross-sectional and case–control studies cannot establish whether *H. pylori* infection precedes endothelial or arterial abnormalities, and reverse causation or bidirectional associations remain possible. Patients with cardiometabolic disease may have different patterns of healthcare access, gastrointestinal investigation, previous antibiotic or acid-suppressive treatment, dietary behavior, nutritional status, and medication use, potentially influencing both the classification of infection and the measured vascular phenotype.

Residual confounding is also likely. Smoking, body mass index and adiposity, hypertension, diabetes, dyslipidemia, renal function, diet, physical activity, socioeconomic disadvantage, and concomitant therapies may be associated with both *H. pylori* exposure and vascular dysfunction. In particular, antihypertensive, lipid-lowering, glucose-lowering, and other cardiovascular therapies may directly affect endothelial function, blood pressure, inflammatory markers, or arterial-stiffness measurements, while previous antibiotics or proton-pump inhibitors may affect the detection of active infection. Adjustment for these variables was inconsistent across studies, and even extensive multivariable models cannot exclude unmeasured or imperfectly measured confounding. Consequently, associations reported as statistically independent should not be regarded as proof of a direct causal vascular effect of *H. pylori*.

## 8. Conclusions

*H. pylori* should be interpreted neither as an incidental serological finding nor as a universal cardiovascular pathogen. The most defensible interpretation is that chronic infection is associated with vascular dysfunction in selected biological contexts and may contribute to it when active infection or strain virulence coincides with a susceptible inflammatory, metabolic, or gastric-disease substrate. However, the predominantly observational evidence, uncertain temporal sequence, and potential for residual confounding preclude causal conclusions.

This model is most persuasive for endothelial dysfunction. Active infection, CagA-dependent exosome signaling, oxidative stress, selective impairment of endothelium-dependent relaxation, and improvement after eradication form a coherent mechanistic and translational sequence linking gastric infection to vascular homeostasis. Evidence for arterial stiffness is less uniform, but becomes more clinically meaningful in populations enriched for early vascular aging, inflammatory activation, cardiometabolic disease, or advanced gastric injury.

The broader implication is that *H. pylori* may contribute to early vascular aging as part of an immunometabolic network linking chronic mucosal infection, endothelial injury, extracellular-vesicle signaling, oxidative stress, and arterial-wall remodeling.

## Figures and Tables

**Table 1:** Clinical evidence on *H. pylori* infection and endothelial or endothelial-adjacent vascular phenotypes.

First Author, Year	Design and Population	Infection Assessment	Vascular Endpoint	Main Findings	Vascular Relevance
Prasad, 2002 [13]	Coronary angiography cohort	IgG antibodies to five pathogens, including *H. pylori*	Intracoronary acetylcholine, sodium nitroprusside, adenosine	Cumulative pathogen burden was associated with coronary artery disease and acetylcholine-mediated endothelial dysfunction, whereas sodium nitroprusside and adenosine responses were preserved.	Selective endothelial dysfunction as the vascular phenotype most closely related to chronic infectious burden.
Khairy, 2003 [3]	Healthy young men without cardiovascular risk factors	Serology for *H. pylori*, C. pneumoniae, CMV, EBV	Brachial FMD	In healthy young men, *H. pylori* seropositivity and total infectious burden were not associated with brachial FMD.	Low-risk serology-based setting with no detectable endothelial signal, consistent with the limited vascular meaning of remote exposure alone.
Grąbczewska, 2006 [26]	Unstable angina	*H. pylori* and C. pneumoniae serology; Western blot antigen profile	vWF, TM, tPA:Ag, PAI-1:Ag, CRP	Selected *H. pylori* antigenic profiles were associated with endothelial or fibrinolytic biomarkers, whereas binary serostatus was less informative.	Antigenic profile as a potentially richer biological exposure marker than simple *H. pylori* seropositivity.
Evrengul, 2007 [27]	Slow coronary flow case-control study	14C urea breath test	TIMI frame count, homocysteine, folate	Slow coronary flow was associated with higher *H. pylori* prevalence, higher homocysteine, lower folate, and increased TIMI frame count.	Possible microvascular and one-carbon-metabolism pathway in selected coronary phenotypes.
Szklo, 2009 [4]	MESA random sample	Antibodies to five pathogens and pathogen burden	CIMT and CAC	In MESA, *H. pylori* seropositivity and pathogen burden were not associated with carotid intima-media thickness or coronary artery calcification.	Major negative structural-vascular study, reinforcing the distinction between endothelial dysfunction and established arterial remodeling.
Rasmi, 2012 [28]	Cardiac syndrome X	Anti-*H. pylori* IgG	Homocysteine	Homocysteine was higher in cardiac syndrome X than in controls, but did not differ according to *H. pylori* status within the syndrome.	Limited support for homocysteine as a universal mediator of *H. pylori*-related vascular dysfunction.
Kurkowska-Jastrzębska, 2016 [30]	Controls, post-stroke, post-MI	Antibodies to *H. pylori* and other pathogens	CIMT, ICAM-1, immune complexes, hs-CRP	In controls and patients after stroke or myocardial infarction, inflammatory and endothelial biomarkers varied by disease stage, while pathogen serology showed limited relation to structural vascular markers.	Disease-stage-dependent biomarker pattern, with weak evidence for a discrete *H. pylori*-related structural vascular signal.
Xia, 2020 [5]	Young adults and mouse model	Biopsy/13C urea breath test; CagA-positive experimental infection	FMD, aortic relaxation, endothelial migration, tube formation, proliferation	Active *H. pylori* infection was associated with reduced FMD in young adults; experimental infection impaired acetylcholine-mediated relaxation while preserving nitroglycerin responsiveness, and endothelial function improved after eradication.	Strong translational evidence for active infection-related, selective, and potentially reversible endothelial dysfunction.
Abdelhai, 2026 [14]	Pediatric endoscopy cohort	Endoscopic confirmation and post-eradication reassessment	Brachial FMD, hs-CRP, lipids, Sydney score	In children, *H. pylori* infection was associated with lower brachial FMD, higher inflammatory and lipid indices, correlation with gastric inflammatory severity, and improvement after eradication.	Early-life endothelial dysfunction linked to active gastric infection and inflammatory burden, with reversibility after eradication.

CAC, coronary artery calcification; CIMT, carotid intima-media thickness; CMV, cytomegalovirus; CRP, C-reactive protein; EBV, Epstein–Barr virus; FMD, flow-mediated dilation; hs-CRP, high-sensitivity C-reactive protein; ICAM-1, intercellular adhesion molecule-1; IgG, immunoglobulin G; MESA, Multi-Ethnic Study of Atherosclerosis; MI, myocardial infarction; PAI-1:Ag, plasminogen activator inhibitor-1 antigen; TIMI, Thrombolysis in Myocardial Infarction; TM, thrombomodulin; tPA:Ag, tissue plasminogen activator antigen; vWF, von Willebrand factor.

**Table 2:** Experimental and translational mechanisms linking *H. pylori* infection to endothelial injury.

First Author, Year	Model	Endothelial Readout	Main Quantitative or Mechanistic Findings	Rescue or Modifier	Mechanistic Relevance
Chi, 2019 [29]	HUVECs and infected mice	Proliferation, migration, tube formation, GATA3, CHI3L1, p38 MAPK	*H. pylori* inhibits proliferation, migration, and tube formation and increases GATA3, CHI3L1, and phosphorylated p38	*GATA3* knockdown restores endothelial phenotype	The GATA3–CHI3L1–p38 MAPK axis identifies a regulated inflammatory pathway through which *H. pylori* may impair endothelial repair.
Xia, 2020 [5]	Young adults, mice, gastric epithelial cells, endothelial cells	FMD, ACh relaxation, endothelial migration, tube formation, proliferation	CagA-containing exosomes enter endothelial cells and impair migration, tube formation, and proliferation	Eradication and GW4869 improve or preserve endothelial function	CagA-containing exosomes provide a plausible biological route from gastric infection to impaired endothelial migration, proliferation, and tube formation.
Xia, 2022 [6]	CagA-positive and CagA-negative infected mice; endothelial-cell exosome exposure	Aortic ROS, ACh relaxation, early atherosclerosis	CagA-positive but not CagA-negative infection increases ROS, impairs ACh relaxation, and enhances early atherosclerosis	N-acetylcysteine and GW4869 prevent endothelial dysfunction	The CagA–exosome–ROS sequence places oxidative stress upstream of endothelial dysfunction and early vascular injury.
Zhang, 2023 [24]	Male and female mice infected with CagA-positive *H. pylori*	ACh relaxation, ROS, endothelial migration, tube formation, proliferation	Male but not female mice develop endothelial dysfunction and increased aortic ROS	N-acetylcysteine prevents endothelial impairment	The male-specific endothelial phenotype supports sex as a modifier of CagA-driven oxidative vascular injury.

ACh, acetylcholine; CagA, cytotoxin-associated gene A; CHI3L1, chitinase 3-like 1; FMD, flow-mediated dilation; GATA3, GATA-binding protein 3; GW4869, exosome-release inhibitor; HUVECs, human umbilical vein endothelial cells; MAPK, mitogen-activated protein kinase; ROS, reactive oxygen species.

**Table 3:** Clinical and contextual evidence linking *H. pylori* infection or gastric injury to arterial stiffness.

First Author, Year	Design and Population	Infection or Gastric Assessment	Stiffness Endpoint	Main Findings	Vascular Relevance
Espinola-Klein, 2000 [17]	Cardiovascular cohort	Serology for *H. pylori* and other pathogens	Carotid elastic pressure modulus, CIMT, stenosis	*H. pylori* antibodies were not associated with carotid elastic pressure modulus, intima-media thickness, or carotid stenosis.	Serology-based exposure in an advanced cardiovascular cohort provides little support for direct *H. pylori*-related arterial remodeling.
Adachi, 2003 [18]	Health-screening cohort	Anti-*H. pylori* IgG	HCPWV, HAPWV, ABI	*H. pylori* seropositivity was associated with lower HDL cholesterol and higher leukocyte count; heart–carotid PWV was higher in seropositive individuals aged <39 years.	Possible early-life vascular signal, detectable before age- and pressure-related arterial remodeling become dominant.
Saijo, 2005 [19]	Japanese cohort	Anti-*H. pylori* antibody and CRP	baPWV	In men, *H. pylori* seropositivity was associated with high baPWV, particularly in the presence of elevated CRP and at age ≤49 years.	Male-specific and inflammation-dependent stiffness pattern, consistent with vascular susceptibility rather than a uniform serological effect.
Ohnishi, 2008 [20]	Type 2 diabetes	Anti-*H. pylori* IgG	PWV	*H. pylori*-positive patients with type 2 diabetes had higher PWV than uninfected patients, and infection remained independently associated with PWV.	Diabetic vascular substrate in which infection may contribute to greater arterial stiffening.
Torisu, 2009 [21]	Older Japanese adults	Anti-*H. pylori* IgG and pepsinogen-defined AG	baPWV and CAVI	Pepsinogen-defined atrophic gastritis was associated with higher baPWV and CAVI, whereas *H. pylori* IgG status alone showed only nonsignificant trends.	Gastric atrophy as a cumulative exposure marker, more informative than antibody status alone in older adults.
Koyama, 2016 [33]	Nested case–control	*H. pylori* serology	PWV and homeostatic markers	*H. pylori* seropositivity predicted cognitive decline in the final stepwise model [OR 4.468; 95% CI, 1.535–13.00], whereas PWV was not independently predictive.	Systemic immune–metabolic consequences of infection not fully explained by measurable arterial stiffness.
Kutluana, 2019 [22]	Endoscopy cohort	Histologic GIM and AG	PWV	Patients with gastric intestinal metaplasia or atrophic gastritis had higher PWV and a higher prevalence of PWV >10 m/s than controls; vitamin B12 deficiency independently predicted elevated PWV.	Advanced gastric injury and micronutrient disturbance as markers of cumulative vascular exposure.
Choi, 2019 [31]	Asymptomatic cohort	Anti-*H. pylori* IgG	CAVI	*H. pylori* seropositivity was independently associated with high CAVI in an asymptomatic cohort.	Population-level CAVI association, compatible with a modest stiffness signal in asymptomatic adults.
Yang, 2020 [23]	Diabetes cohort	13C urea breath test	baPWV and 10-year CV risk	In diabetes, active *H. pylori* infection was associated with higher baPWV, more severe peripheral arterial stiffness, and higher estimated 10-year cardiovascular risk.	Active infection in diabetes as a clinically enriched stiffness phenotype.
Krupa, 2021 [34]	Guinea-pig infection and high-fat diet model	Experimental infection and diet	Pulse-wave amplitude, endothelial inflammation	In an experimental model, *H. pylori* infection combined with a high-fat diet increased inflammatory activation and reduced pulse-wave amplitude.	Experimental support for infection–diet synergy in endothelial inflammation and impaired arterial elasticity.
Choi, 2022 [12]	NAFLD/MAFLD cohort	Anti-*H. pylori* IgG	CAVI	NAFLD or MAFLD combined with *H. pylori* infection conferred higher odds of increased CAVI than metabolic liver disease alone.	Additive hepatic–metabolic and infectious burden in arterial stiffening.
Kim, 2025 [25]	Young-adult histology cohort	Histologic infection and Updated Sydney System score	ePWV	Histologically confirmed *H. pylori* infection was associated with higher ePWV; severe infection showed the strongest association with elevated ePWV.	Severity-gradient evidence linking histologic gastric infection to early arterial stiffening.

ABI, ankle–brachial index; AG, atrophic gastritis; A/G, albumin-to-globulin ratio; baPWV, brachial–ankle pulse-wave velocity; CAVI, cardio–ankle vascular index; C-CVD, cardio–cerebrovascular disease; CI, confidence interval; CIMT, carotid intima-media thickness; CRP, C-reactive protein; CV, cardiovascular; ePWV, estimated pulse-wave velocity; FRS, Framingham risk score; GIM, gastric intestinal metaplasia; HAPWV, heart–ankle pulse-wave velocity; HCPWV, heart–carotid pulse-wave velocity; HDL, high-density lipoprotein; ICVD, ischemic cardiovascular disease; IgG, immunoglobulin G; MAFLD, metabolic dysfunction-associated fatty liver disease; MetS, metabolic syndrome; NAFLD, nonalcoholic fatty liver disease; OR, odds ratio; PWV, pulse-wave velocity.

## Data Availability

No new data were created or analyzed in this study. Data sharing is not applicable to this article.

## Abbreviations

The following abbreviations are used in this manuscript:

ABI	Ankle–brachial index
ACh	Acetylcholine
AG	Atrophic gastritis
A/G	Albumin-to-globulin ratio
baPWV	Brachial–ankle pulse-wave velocity
CagA	Cytotoxin-associated gene A
CAC	Coronary artery calcification
CAD	Coronary artery disease
CAVI	Cardio–ankle vascular index
C-CVD	Cardio–cerebrovascular disease
CHI3L1	Chitinase 3-like 1
CI	Confidence interval
CIMT	Carotid intima-media thickness
CMV	Cytomegalovirus
COVID-19	Coronavirus disease 2019
CRP	C-reactive protein
CV	Cardiovascular
EBV	Epstein–Barr virus
ePWV	Estimated pulse-wave velocity
FMD	Flow-mediated dilation
FRS	Framingham risk score
GATA3	GATA-binding protein 3
GIM	Gastric intestinal metaplasia
GW4869	Exosome-release inhibitor
HAPWV	Heart–ankle pulse-wave velocity
HCPWV	Heart–carotid pulse-wave velocity
HDL	High-density lipoprotein
*H. pylori*	Helicobacter pylori
hs-CRP	High-sensitivity C-reactive protein
HUVECs	Human umbilical vein endothelial cells
ICAM-1	Intercellular adhesion molecule-1
ICVD	Ischemic cardiovascular disease
IgG	Immunoglobulin G
IS	Ischemic stroke
MAFLD	Metabolic dysfunction-associated fatty liver disease
MAPK	Mitogen-activated protein kinase
MESA	Multi-Ethnic Study of Atherosclerosis
MetS	Metabolic syndrome
MI	Myocardial infarction
NAFLD	Nonalcoholic fatty liver disease
OR	Odds ratio
PAI-1:Ag	Plasminogen activator inhibitor-1 antigen
PWV	Pulse-wave velocity
ROS	Reactive oxygen species
TIMI	Thrombolysis in Myocardial Infarction
TM	Thrombomodulin
tPA:Ag	Tissue plasminogen activator antigen
vWF	von Willebrand factor
